# Medical laboratory accreditation status and associated factors in selected private and government health facilities of Addis Ababa, Ethiopia

**DOI:** 10.11604/pamj.2023.45.96.29164

**Published:** 2023-06-21

**Authors:** Meseret Tesema, Abay Sisay

**Affiliations:** 1 Ethiopian National Accreditation Office (ENAO), Addis Ababa, Ethiopia,; 2 Addis Ababa University, College of Health Sciences, Department of Clinical Laboratory Sciences, Addis Ababa, Ethiopia

**Keywords:** Accreditation, ISO 15189, Addis Ababa, Ethiopia

## Abstract

**Introduction:**

quality medical laboratory service(s) is a key to patient safety with a great emphasis on medical diagnoses and treatment. ISO 15189 laboratory accreditation is an effective way to demonstrate competency. Despite the benefits, there are considerable exigent efforts towards achieving its target, mainly in sub-Saharan Africa. Hence, determining those factors that hinder laboratory quality services and the process of accreditation is important to address and resolve. Thus, this study aimed to assess medical laboratory accreditation process and in selected private and government health facility laboratories in Addis Ababa, Ethiopia.

**Methods:**

institutional-based cross-sectional study design was conducted in Addis Ababa from July 1 to August 30, 2018. Data was entered into EPI-data version 3.1 and analyzed by SPSS version 23. Data from focus group discussions were categorized and discussed thematically. Additionally, logistic regression analyses were computed to examine the relationship between the explanatory and response variable.

**Results:**

a total of 411 professionals participated in this study, of which 117(28.8%) participants were female, 280 (68.2%) participants with a bachelor´s degree, and 352 (85.6%) participants had information about accreditation. The current laboratory accreditation status in Addis Ababa is 3.6%. The primary identified factors were gaps related to method verification/validation, equipment calibration, and continual program quality improvement.

**Conclusion:**

strengthening laboratory management standards towards accreditation (SLMTA) will significantly improve the accreditation process. However, there are internal and external factors may hinder the current accreditation process. Therefore, all responsible agencies/services should give more attention to solving those identified major barriers to achieving accreditation.

## Introduction

Laboratory accreditation is a formal recognition by a qualified third party to carry out specific services and are tasked to produce “accurate measurable results” within acceptable industry limits, consistently and sustainably [1,2]. Medical laboratories serve as key partners in patient safety, and influence 70% of medical diagnoses [3]. Further defining these services will help to combat major infectious diseases [4]. To get accredited, a medical laboratory has to comply with all the requirements of the international standard ISO 15189 [5]. The improvement of the quality of testing services in medical laboratories is a high priority in many countries [6]. Ethiopia is one of the sub-Saharan African countries where laboratory infrastructure and quality assurance activities remain weak [7]. To address this issue, the country implemented the WHO-AFRO Stepwise Laboratory Quality Improvement Process, SLIPTA/SLMTA (2009) to help laboratories comply with the ISO 15189 standard [8,9]. Implementation of laboratory standards is verified through the process of accreditation. However, achieving practical and sustainable laboratory accreditation and securing patient safety is a major challenge generally due to a lack of leadership, attention to detail, lack of resources, and commitment to excellence. Therefore, accreditation standards must encourage improved performance, while at the same time be achievable and not overly prescriptive [10,11]. Even though Ethiopian National Accreditation Office (ENAO) which was established in 2012 and achieved full recognition, by peer-evaluation, the International Laboratory Accreditation Co-Operation (ILAC) on Medical and Testing Laboratory Accreditation Service in October 2017, noted that the number of accredited facilities remain very low. Additionally, there is no research data regarding accreditation services in Ethiopia. Moreover, there is little information available on the status of laboratories´ progress toward the accreditation process [12]. Thus, this study was conducted to assess the current status of accreditation and identify the associated factors in selected private and government health facility laboratories of Addis Ababa, Ethiopia, and provide baseline information to policy and decision-makers to work on their challenges.

## Methods

### Study design, setting and period

An institutional-based cross-sectional study design using qualitative and quantitative data collection approach was used to assess medical laboratory accreditation status and associated factors in selected private and government health facility laboratories in Addis Ababa Ethiopia and was conducted from July 1^st^ to August 30^th^, 2018. A self-administered, semi-structured questionnaire with focus group discussion and in-depth interviews was applied. Addis Ababa is the capital city of Ethiopia. It is the biggest city in the country and a chartered city having three layers of government namely, city government at the top, 10 sub-cities in the middle and 116 Woreda. There are 6 regionals, 5 federal (including one university hospital), 2 non-governmental organizations supported, 30 private, 1 defense, 1 prison, and 1 police hospital laboratory. There are also 100 (currently functional) government and 4 NGO-supported health centers; 7 Public, 500 private, 31 NGO-supported clinics, and 30 private hospitals [13,14]. The study was conducted in selected private and government health facilities laboratories that had implemented SLIPTA/SLMTA and/or ISO 15189 accreditation. In Addis Ababa there are 92 health centers, 6 government hospitals, and 5 private health laboratories that participated in the SLMTA program and the study was conducted with these selected facilities.

### Sample size determination and sampling procedure

A mixed sampling technique was applied by a purpose sampling technique to select the health facility laboratories from the total health facility laboratories in Addis Ababa, based on their SLIPTA/SLMTA and/or ISO 15189 implementation. The laboratory Department Head, the Quality Officer, the Medical Director, and the Chief Executive Officer (CEO) were primarily targeted and the remainder were selected based on a simple random sampling by the data collector. The accredited facilities received one questionnaire which was distributed to the Quality Officers and Facility Managers regarding the accreditation implementation process, considering their responsibilities. A single population proportion formula was used to determine the sample size, and considered the following assumptions: due to the absence of reliable previous study data that represented the laboratory professionals´ knowledge of quality systems and accreditation, a selected proportion of 50% was taken considering the level of significance = 0.05, marginal of error (d) = 5%, sample size = n, Z (a/2) = Z-score at 95% confidence interval = 1.96. The calculating sample size (n) is as follows: N=Za/22P (1-p)/d2; n=1.962*0.5*0.5/0.052 = 384 then n=384. Then, by adding 10% of the non-response rate, we arrived at 423 professionals as the final sample size.

Based on the profile of the health institution laboratory professional, on average there are 6 laboratory professionals in the health center and 20 laboratory professionals in the hospital. In Addis Ababa, there were 92 health centers, 6 government hospitals, and 5 private health laboratories that participated in the SLMTA program. In each of the health centers, there are six laboratory professionals, which accounts for 552 (92 * 6), whereas there are 20 professionals in the hospitals, which accounts for 120 (6*20), and 5 professionals working in private health facilities, which accounts for 25 (5*5). Hence, a total of 697 laboratory professionals are available in Addis Ababa working in the SLMTA implemented facilities. The number of questionnaire respondent professionals taken from each laboratory was calculated using the probability proportional sampling technique. Thus, from each of the facilities, the sample group was taken by multiplying the number of all laboratory professionals in a given laboratory group by the ratio of a total sample size to total laboratory professionals, which is (384/697=0.5509) as a multiplying factor. By this calculation, we come up with 304, 66, and 14 questionnaire respondents from health centers, hospitals, and private health facilities respectively. Then we added a 10% non-respondent rate resulting in 334 from the health centers, 73 from Hospitals, and 16 from private health facilities. The sample size for the qualitative part was selected purposively and included the Laboratory Head, Quality Officer, the Medical Director and the CEO for an in depth interview of each laboratory for focus group discussion.

### Data collection, quality assurance, data management, and analysis

**Data collection and data quality assurance**

Data was collected using self-administered semi-structured questionnaires, in-depth interviews and focus group discussions. The semi-structured questionnaire was developed to collect objective evidence from participants by referring to the horizontal checklist of ISO 15189: 2012 standard, related articles and various recent guidelines and other documents regarding accreditation [4,9]. It was refined following pre-testing, by removing the undecided part from options along with adding risk assessment and training on the measurement of uncertainty. The questionnaire consisted of four parts which included socio-demographic, knowledge, attitude, and practice questions. It was pre-tested at St. Peter Hospital, a federal hospital, to evaluate its clarity and applicability according to the objective of the study. Quantitative data was collected from all study participants using a semi-structured questionnaire. Qualitative data from Laboratory Heads, Technical Staff, Safety Officers, Medical Directors and Quality Officers were obtained via focus group discussions and in-depth interviews with associated CEOs. Trained Data Collectors were deployed for the data collection by structured questionnaire, whereas the qualitative data from laboratory heads, quality officers and medical directors were collected by the research principal investigator to ensure relevant data was obtained. Data were collected by trained and well-experienced personnel under proper supervision and oversight. Each of the data collectors has a diploma and/or degree in the field of Medical Laboratory Science. The Principal Investigator rechecked the completeness and clarity of each questionnaire. Each questionnaire was given a different identification number and was validated by double data entry (Annex 1).

### Data management and analysis

Before doing the analysis, the entire data field was cross-checked for reliability and completeness on both the collected hard copy and soft copy of the data entered. After reviewing for completeness, coding was performed by the Principal Investigator and followed by data entry into EPI data Version 3 and SPSS version 23 software (IBM Corporation, Chicago, IL, USA) for cleaning and analysis. Descriptive statistical values including frequencies, percentages, mean and standard deviations were used primarily to summarize results as well as describe the data. Cross tabulations were used to determine if there is an insufficient cell count within each of the variable categories. The crude and adjusted odds ratio (COR&AOR) with confidence intervals (CIs) and p-values from Wald F statistics was used to determine the statistical and significant associations b/n implementation of accreditation and demographic variable. Bi-variate and Multi-variate logistic regression analyses were computed to examine the relationship b/n the explanatory variables and response variables. The qualitative data from focus group discussions and in-depth interviews were categorized and discussed by using thematic method analysis using open code software. To protect from data manipulation, the data was stored in a password-protected computer, and backup was saved onto a flash drive and CDs.

### Ethical approval and consent to participate

Ethical clearance was obtained from GAMBY Medical and Business College and a formal official Letter of Cooperation (LOC) was written to the Addis Ababa Health Bureau followed by an ethical approval and LOC for each sub-city and hospitals from Addis Ababa Health Bureau. Written consent was obtained from each participant prior to conducting the study. Additionally, confidentiality was assured for all the information provided and personal identifiers were not included in the report.

## Results

### Socio-demographic characteristics of respondents

A total of 411 professionals (Laboratory Managers, Quality Officers, Technical Staff, and CEOs) participated in this study. 334 were from health centers, 73 were from hospitals, and 16 were from private health facilities. 117 (28.8%) were female, 239 (56.5%) of respondents were between 26-30 years old and 289 (68.3%) participants had a bachelor´s degree, as illustrated in Table 1.

**Table 1:** socio-demographic characteristics of laboratory professionals working in selected government and private health institutions in Addis Ababa, Ethiopia (n=411), 2018

Characteristics	Frequency	Percent
**Gender**		
female	117	28.8
male	294	71.2
**Age of the participant**		
18 - 25	2	0.5
26 - 30	235	56.5
31 - 35	106	25.5
36 - 40	41	10.2
41 - 45	19	5.0
>45	08	2.4
**Educational level**		
Diploma or below	44	11.3
First degree (BSc)	285	68.3
Second degree (MSc)	82	20.3
**Monthly income in ETB**		
2000-3500	23	5.9
3500-5500	94	22.7
5,500-7500	151	36.4
7,500-9500	115	27.9
>9,500	31	7.8
**Work experience**		
1-4 years	47	11.8
5-10 years	280	66.9
11-15 years	37	9.5
>15 years	47	11.8

### Knowledge and attitude on accreditation

Of the 411 respondents, 85.8% had information about accreditation and 285 (68.6%) had received training related to ISO 15189 standards. 103 respondents were from SLIPTA/SLMTA implemented health facilities with only 3.3% of laboratories having achieved accreditation and 96.7% were non-accredited facilities as detailed and depicted in Table 2. The majority of respondents (91.3%) believed that accreditation is important and that ISO 15189 training is helpful for implementation (98.3%). 68.8% had maintained practice of LQMS and 17.7% had reverted and suspended the practice of LQMS. 76.1% of respondents believed that being in a non-accredited laboratory affected client satisfaction, as detailed in Table 3.

**Table 2:** knowledge on accreditation laboratory professionals working in government and private health institutions in Addis Ababa, Ethiopia (n=411), 2018

No	Variable	Frequency		Percent	
		Yes	No	Yes	No
1	Information on accreditation	357	54	86.8	13.2
2	Training related to ISO 15189	285	126	69.3	30.6
3	Advisory/ consultants/ mentors	181	230	44	56
4	Information on accreditation body found in Ethiopia	344	67	83.6	16.4
5	Laboratory Accredited	15	396	3.6	96.4

**Table 3:** attitude on Accreditation among laboratory professionals working in government and private health institutions in Addis Ababa, Ethiopia (n=411), 2018

No	Variable	Agree		Disagree	
		Frequency	%	Frequency	%
1	Believe accreditation is important	386	91.3	37	8.7
2	ISO 15189 training is helpful for implementation of accreditation	416	98.3	7	1.7
3	Implementation of LQMS	348	82.3	75	17.7
4	Non-accredited laboratories affect client satisfaction	322	76.1	101	23.9

**Factors associated with low implementation of accreditation:** we received information from respondents with identifying factors that affect implementation of accreditation where an absence of training on ISO 15189 standard 133 (31%), insufficient management support, 214 (5.2%) and lack of equipment calibration traceability NMI/IBM (85.1%) were all factors as the detailed in Table 4.

**Table 4:** factors associated with the low implementation of accreditation reported by laboratory professionals working in selected government and private health institutions of Addis Ababa, Ethiopia, (n=411), 2018

Questions	Yes		No	
	Frequency	Percentage	Frequency	Percentage
Competent staff turnover	188	45.9	223	54.1
Sufficient Resources	146	36.6	265	63.4
Top management support	197	48	214	52
Equipment full information	244	59.1	167	40.9
Perform equipment calibration	280	67.6	131	32.4
Information availability accreditation body in Ethiopia	342	82.3	69	17.7
Backup power supply	319	76.8	92	23.2
EQA participation	190	46.3	221	53.7
Calibrator reagents traceability to IBM	96	24.1	315	75.9
Staff motivation	132	32.6	279	67.4
IQC for each test	160	39.2	251	60.8
Conduct method validation/ verification	17	5.4	394	94.6
Training on method verification	40	1.9	331	98.1
Calibrated equipment maintains metrological traceability	57	14.1	354	85.1
Selecting and evaluating referral laboratories	342	82.3	69	17.7
List of selected and approved suppliers	327	78.7	84	21.3
continual professional development training on LQMS	45	2	326	98
Conduct risk assessments	88	22.2	323	77.8
Documented contingency plans	352	84.4	59	15.4

### Findings from logistic regression analysis

Multivariate analysis was performed between different factors with ISO 15189 laboratory accreditation. 9 (64.3) respondents who performed method validation/verification regularly in their laboratory were 3.45 times more likely to have a positive effect on laboratory accreditation than laboratories performing incomplete and irregular performance of method verification/validation [AOR=3.4595% CI (2.73 - 31.63)]. Laboratories that have a routine laboratory equipment calibration program with metrological traceability to institutions like NMI traceability were 9.47 times more likely to be accredited than those laboratories that were not performing laboratory equipment calibration testing [AOR=9.47 95%CI (1.75-20.65)]. Among participants of this study, those laboratories that had good practices and knowledge of continual professional development training such as specific training on LQMS showed greater motivation to become accredited than those laboratories that don´t have such regular practices and continual professional development programs [AOR=0.76 95% CI (0.02-0.93] (Table 5).

**Table 5:** factors associated with ISO 15189 laboratory accreditation in Addis Ababa, Ethiopia, 2018

Variables		Accreditation Status		COR,95%CI	AOR,95% CI	P-value
		Yes	No			
Equipment calibration	Perform	8(57.10)	49(13.9)	1	1	
	Not Perform	6(42.9)	348(86.1)	0.15 (0.07-0.31)	9.47(1.71-20.65)**	0.004
Educational Status	Diploma	1(7.2)	43(11.5)	1	1	
	BSc degree	7(50)	281(69.7)	0.03 (0. 01-0. 05)	1.43(1.0-2.01)	
	MSC degree and above	6(42.8)	73(18.8)	0.08(0. 034-0.18)	0.3 (0.87-12.63)	0.079
Continual Improvement Training	LQMS	13(92.8)	375(93.2)	0.32(0.26-0.38)	0.76 (0.02-0.93) **	0.001
	Routine	1(7.2)	22(6.8)	1	1	
Method validation /verification	Perform	5(35.7)	55(15)	1	1	
	Not Perform	9(64.3)	342(85)	0.18(0.07-0.29)	3.45(2.73 -31.25)**	0.001
Experience	1-4	1(7.2)	62(16.1)	1	1	
	5-10 working experience	9(64.3)	253(62.8)	3.87(1.39-5.94)	2.76(1.29-5.72)	
	>10 years’ experience	4(28.6)	82(21.1)	1.38(0.84-3.21)	1.02 (0.45-2.31)	0.96
Monthly Income	≤3500	1(7.2)	24(5.9)	1	1	
	3500-5500	6(42.8)	244(59.6)	2.289(0.540-3.108)	1.103(.620-3.665)	0.37
	5500-7500	7(50)	141(34.5)	0.290(0.178-0.348)	0.273(0.256-0.540)	

### Focus group discussion

Three focus group discussions were conducted. The first group had 6 members (P1-P6), the second group had 7 members (P7-P13) and the third group had 7 members (P14-P20). The facilities selected for FGD were based on their SLIPTA implementation status (high score star 4 and star 1) and accreditation status. From the FGD findings, a majority of the laboratories had information on accreditation since they practice the SLMTA/SLIPTA approach, and all participants(P1-P20) believed that the test results from an accredited laboratory was acceptable everywhere. As one of the determinants of the implementation of accreditation, 70% (P1, P2, P3, P4, P5, P6, P10, P12, P14, P16, P17, P18, P19, and P20) of the participants raised the issue of supplies interruption, resource shortages and instability of supplier´s prices in the market.

Almost half of the respondents (P1, P3, P4, P7, P8, P10, P 15, P18 & P19) indicated “there is a lack of staff motivation due to different reasons” …., such as, top management did not understand how time-consuming the accreditation system implementation was and that sufficient resources were required. In addition, “…most of the activities were the responsibility of the Quality Manager and Laboratory Head resulting in a lack of a uniform understanding of the process and affected staff motivation. Staff turnover also affected the implementation of the accreditation process when specific people involved in the activities and system implementation resigned and progress was stalled”. Another reason raised was “…top managers often did not recognize the overall efforts of their laboratory personnel in accreditation system implementation…”. Another factor raised by the groups was equipment service maintenance was a primary challenge to performing their routine tasks. “Equipment often does not have service maintenance according to the manufacturer´s instructions resulting in test results not being delivered within their stated turnaround time due to the regular occurrence of equipment failure”.

Another factor raised was the availability and cost of proficiency testing, which was often the reason that “… laboratories would only apply for accreditation for a limited scope”. During the focus group discussion “… only a few laboratories participating in proficiency testing from One World Accuracy were applying for accreditation”. Some of the respondents indicated that “… accreditation is not the primary issue rather the need to focus on the fulfillment of the basic supplies was a higher priority”. Some of the participants criticized the purchasing system “…with fluctuation of price as one of the major factors impacting implementation of accreditation”.

### In-depth interview

The in-depth interview was conducted with 16 CEOs of selected private and government health facilities. All the respondents had information on accreditation and were aware of the availability of an accreditation body in Ethiopia. However, some of them did not fully understand the benefits and consequences of the implementation of accreditation nor did they have awareness of training on accreditation. The majority of the respondents raised concerns over inconsistency of supplies in the market, saying “were not always able to access the supplies based on the specification requirement, as frequently reagent stock had run out”. Some of the CEOs raised “lack of staff motivation, resource shortage, and high staff turnover” as the main factors that affected the implementation of accreditation. Some of the CEOs also raised the issues related to the unrecognized benefits of a facility that was accredited. There was a lack of awareness in the community of the difference between an accredited and non-accredited laboratory which impacted motivation to implement accreditation programs. They recommended the government should develop policy relating to the benefits of accreditation.

## Discussion

The present study shows that 389 (85.6%) of respondents had information about accreditation. Current statuses of laboratory accreditation in Addis Ababa is 3.6% even though the majority of respondents have a positive attitude on the importance of accreditation through the acceptance of the reported test result throughout the world, however, they did not have any intentions for personal use, which is a concordant finding with Girma *et al*., 2017 [15]. The other major factor that affected the status of accreditation in Addis Ababa Ethiopia was lack of training on ISO 15189, accounting for 68.9% of the group response. It was agreed that accreditation requires trained and experienced laboratory personnel to lead and be involved in the accreditation process, which is in line with concurrent research performed in the Caribbean Region as ensuring a sufficient number of well-qualified laboratory workers is an ongoing challenge, exacerbated by high levels of attrition as staff exit the public sector for more lucrative jobs in the private sector, either locally or overseas [10]. The major factors that affected the quality of laboratory services were associated with poor human resource management, poor resources provision, poor management commitment, ineffective communication system, and lack of a well-established quality management system, which is similar in findings where services had the lack of adequate resources referenced by Abay Sisay and Eyob *et al*. [4,7].

More than half of accredited facilities were initially approved through their assigned advisor or consultants, while currently, 58.9% do not have access to advisory/consultant resources. Sixty-four point one percent (64.1%) of respondents believed that their laboratories did not have sufficient resources due to a variety of reasons including fluctuation of market prices and currency instability. The majority of the respondents indicated that there was low management support for accreditation. One hundred and seventy-five (175) respondents (41.1%) did not have the required equipment information nor service maintenance requirements, which is similar findings with Abay *et al*. and Nzabahimana *et al*. [4,16]. With the exception of accredited laboratories, many laboratories are not performing equipment calibration traceability checks by the National Methodology Institute, NMI (85.1%). Lack of motivation of laboratory professionals is seen as the major challenge for the success of laboratory accreditation which is a similar finding to Girma *et al*. and Nicola´s report [15,17]. Risk management was the major change in the new version ISO15189, 2012 of the standards. It was stated that the role of the Laboratory Director is to ‘design and implement a contingency plan’ and that these plans should be tested periodically. It also addresses focusing on the risks of possible failures of test results; noting everything must be done to reduce and/or eliminate these risks in the event of failure or downtime. However, this was only undertaken in the accredited laboratories of Addis Ababa, Ethiopia [18].

According to the findings of this study, 94% of the respondent laboratories were not performing method validation/verification due to a lack of training on method validation/verification and measurement of uncertainty. ISO 15189 requires verifying the stated validation/performance of all the examination procedures. When the validation data from the manufacturer is incomplete, unsuitable, or has been obtained with unclear procedures, the laboratory staff may find it difficult to verify or even understand the information. The laboratory professionals should be able to clearly evaluate manufacturers´ information usually which is usually contained in the technical (package) insert. In particular, they need to establish (I) the quality specifications for each test according to its inherent characteristics, (II) the criteria of adequateness used for establishing performance characteristics of examination procedures during validation, and (III) stringent quality specifications needed for purchasing analyzers and reagents. This assists in guaranteeing the availability of diagnostic systems with the highest possible metrological traceability and the most accurate information about performance characteristics of examination procedures derived from official documents and guidelines [19].

Due to the absence of a proficiency test provider in the country, almost half of the respondent did not participate in EQA. What the laboratory staff referred to as inter-laboratory trials were really result confirmation with a reference laboratory, as reported by Ashebir *et al*. [20]. Uniform awareness of all laboratory staff was relevant for the sustainability of accreditation implementation and not simply affected by staff turnover. If the system is established and all staff members had confirmed competency on the implementation of the system, then this can reduce the number of with drawls of accredited facilities and maintain the system consistence which is similar to the findings of Girma *et al*. and Thiha S *et al*. [15,21].

## Conclusion

Most of the factors affecting laboratory accreditation were similar regardless of the type of health facilities and also majority of the laboratories had information about accreditation and understood the ultimate goal of the process which is accreditation. However, there were identified factors affecting the achievement of accreditation. This study demonstrated that the majority of the factors centered around the commitment from top management, suitably trained laboratory personnel, and creating an awareness of the process and benefits of achieving accreditation. Some facilities need government involvement to resolve their issues. By raising staff awareness of accreditation and increasing staff motivation with a uniform competency approach, it is possible for a laboratory to achieve accreditation in at least one specific scope and to work towards establishing an effective accreditation system throughout the laboratory. Hence, all responsible managers should focus attention to solving those identified major factors towards achieving accreditation.

### What is known about this topic

- *Benefit of ISO15189 laboratory accreditation for assuring the quality of laboratory results;*
- *Medical laboratories accreditation as a key indicator in patient safety, by which results influence 70% of medical diagnoses*.

### What this study adds

- *The quality of laboratory services was more affected by poor human resource management, poor resources provision, poor management commitment, ineffective communication system, and lack of a well-established quality management system;*
- *Laboratory facilities performing a method validation/verification regularly were 3.45 times more likely to have a positive effect on laboratory accreditation than laboratories performing incomplete and irregular performance of method verification/validation;*
- *Professional competency is recognized as a strong contributor to Successful implementation of ISO 15189: 2012 laboratory accreditation*.

## Annex

**Annex 1**: supplementary files (PDF 394 KB)
